# Trail making test performance in youth varies as a function of anatomical coupling between the prefrontal cortex and distributed cortical regions

**DOI:** 10.3389/fpsyg.2014.00496

**Published:** 2014-07-01

**Authors:** Nancy Raitano Lee, Gregory L. Wallace, Armin Raznahan, Liv S. Clasen, Jay N. Giedd

**Affiliations:** ^1^Child Psychiatry Branch, Intramural Research Program, National Institute of Mental Health, NIHBethesda, MD, USA; ^2^Department of Speech and Hearing Sciences, George Washington UniversityWashington, DC, USA

**Keywords:** executive function, anatomical covariance, cortical thickness, magnetic resonance imaging, Trail Making Test, brain, child, adolescent

## Abstract

While researchers have gained a richer understanding of the neural correlates of executive function in adulthood, much less is known about how these abilities are represented in the developing brain and what structural brain networks underlie them. Thus, the current study examined how individual differences in executive function, as measured by the Trail Making Test (TMT), relate to structural covariance in the pediatric brain. The sample included 146 unrelated, typically developing youth (80 females), ages 9–14 years, who completed a structural MRI scan of the brain and the Halstead-Reitan TMT (intermediate form). TMT scores used to index executive function included those that evaluated set-shifting ability: Trails B time (number-letter sequencing) and the difference in time between Trails B and A (number sequencing only). Anatomical coupling was measured by examining correlations between mean cortical thickness (MCT) across the entire cortical ribbon and individual vertex thickness measured at ~81,000 vertices. To examine how TMT scores related to anatomical coupling strength, linear regression was utilized and the interaction between age-normed TMT scores and both age and sex-normed MCT was used to predict vertex thickness. Results revealed that stronger Trails B scores were associated with greater anatomical coupling between a large swath of prefrontal cortex and the rest of cortex. For the difference between Trails B and A, a network of regions in the frontal, temporal, and parietal lobes was found to be more tightly coupled with the rest of cortex in stronger performers. This study is the first to highlight the importance of structural covariance in in the prediction of individual differences in executive function skills in youth. Thus, it adds to the growing literature on the neural correlates of childhood executive functions and identifies neuroanatomic coupling as a biological substrate that may contribute to executive function and dysfunction in childhood.

## Introduction

For over 150 years, scientists studying cognition have noted the important role of the frontal lobes in the regulation of behavior and cognition (see Braver and Ruge, 2006 for a review). While early investigations focused mainly on adult clinical populations, more recent research has described the protracted development of both executive functions and the frontal lobes within the context of normative development.

Executive function is an umbrella term referring to a collection of skills (such as working memory, planning, inhibition, and cognitive flexibility) that are thought to be essential for solving unfamiliar problems and coping with changing demands in one's environment (Lezak et al., 2004). Normative studies indicate that executive function skills develop across childhood and into early young adulthood, with different skills reaching “mature” adult levels at different points in development.

Studies of the protracted nature of the development of executive functions within the context of typical development span several decades. Starting with the early work of Welsh and Pennington (1988) and continuing to more recent investigations (Hooper et al., 2004; Luciana et al., 2005; Huizinga et al., 2006; Conklin et al., 2007), a large corpus of data now exists documenting that youth continue to make gains in performance on several different executive function tasks into the mid to late teens (Luna et al., 2004; Luciana et al., 2005).

Complementing these behavioral studies, morphometric studies of the developing brain using structural magnetic resonance imaging have suggested that the prefrontal cortex, thought to be central to the executive functions, is among the latest maturing regions of the brain (e.g., Gogtay et al., 2004). Its protracted development contrasts with the relatively early development of brain regions thought to contribute to more basic sensory and motor functions, such as the somatosensory cortex.

Thus, both behavioral and anatomical data suggest that childhood and adolescence are times in which studies of the anatomical correlates of executive abilities may be most informative in augmenting our understanding of how higher-level cognitive abilities develop typically and atypically. With regard to atypical executive development, most, if not all, developmental disorders are characterized by executive deficits. Examples include attention-deficit/hyperactivity disorder, conduct disorder, autism spectrum disorders, and intellectual disability, to name a few (for reviews, see Pennington and Ozonoff, 1996; Zelazo and Muller, 2002). Furthermore, many psychiatric disorders that develop in late adolescence or early adulthood, such as schizophrenia and depression, are characterized by executive deficits (Orellana and Slachevsky, 2013; Snyder, 2013).

Understanding the neuroanatomical correlates of executive abilities within the context of typical development may inform research seeking to identify mechanisms that contribute to the atypical development of executive functioning in childhood or in disorders that first manifest in adolescence/early adulthood. In the current investigation, we focus on the neuroanatomical correlates of a commonly-used measure of executive function, the Trail Making Test (TMT), in a sample of typically-developing youth, ages 9–14 years. The TMT, like many neuropsychological assessment tools, was first developed for adult populations. The original task, called the Pathways Test, was included in the Army Individual Test of General Ability in the 1940s (Partington and Leiter, 1949). The TMT is probably best known as being a part of the Halstead-Reitan neuropsychological battery (Reitan and Wolfson, 1993). More recently, modified versions of the TMT have become available, such as the Comprehensive Trail Making Test (Allen et al., 2012a) and the Trail Making subtest on the Delis-Kaplan Executive Function System (Fine et al., 2011; Allen et al., 2012b).

Here we utilize the Intermediate form of the TMT from the Halstead-Reitan neuropsychological test battery (Reitan and Wolfson, 1993). This form has two conditions. The first condition, *Trails A*, requires youth to connect fifteen encircled numbers in order, from 1 to 15, as quickly as possible. The second condition, *Trails B*, requires youth to alternate between connecting numbers and letters in order (i.e., 1-A-2-B and so on) as quickly as possible for a total of 15 connections. Performance on both Trails A and Trails B is thought to tap attention, psychomotor speed, and sequencing abilities. In addition, Trails B is thought to assess set-shifting, a commonly recognized executive function that requires individuals to switch their attention between two rules or tasks (Miyake et al., 2000). Often, investigators interested in studying the more “executive” components of the TMT focus on the difference in completion time for Trails B and Trails A. This difference is thought to partially account for the influence of baseline motoric speed or more basic cognitive abilities on performance and instead focus on the increased higher-order executive demands placed on participants during the Trails B condition, namely set-shifting. We will examine the neural correlates of this score (Trails B – A) as well as Trails B time directly.

The vast majority of studies examining the neural correlates of the TMT have been conducted with adults. To the best of our knowledge, only one study (Tamnes et al., 2010) has examined brain-behavior relations in typical youth using structural MRI and the TMT. Like the current study, these researchers utilized cortical thickness as their neuroanatomic phenotype; however, they directly correlated cortical thickness and TMT performance. As will be described in further detail below, our study examines how the coupling of cortical thickness values across the cortex vary as a function of TMT performance. Thus, the two studies use different analytic techniques to examine brain-behavior relations. In their study, Tamnes and colleagues examined cortical thickness and executive function correlations using the TMT and several other tasks. Surprisingly, the authors reported that most significant correlations between executive function task performance and cortical thickness were found in posterior brain regions. Only one task, another measure of set-shifting, called Plus Minus, was associated with precentral gyrus thickness. Thus, this study highlights the importance of non-frontal regions in accounting for individual differences in executive function in a pediatric sample.

Because of the scarcity of studies examining the neuroanatomic correlates of TMT or set-shifting in youth, we will turn to the adult literature to help generate hypotheses for our study. These investigations include studies of patients with lesions in different anatomic locations as well as both structural and functional magnetic resonance imaging (sMRI and fMRI, respectively) studies within the context of health, aging, and psychiatric illness. With regard to lesion studies, there is a large corpus of research implicating the frontal lobes in the completion of set-shifting tasks, including the TMT (Eslinger and Grattan, 1993; Stuss et al., 2001; Aron et al., 2004; McDonald et al., 2005; Yochim et al., 2007). However, the importance of the frontal lobes to task performance does not appear to be specific, as studies of patients with non-frontal lesions also demonstrate impairment on the TMT. In fact, a meta-analysis demonstrated that while frontal patients showed a small but statistically significant disadvantage on Trails A relative to patients with non-frontal lesions, a statistically significant disadvantage was *not* found for Trails B, as would be expected (Demakis, 2004). Thus, it is clear from this meta-analysis that damage to other brain regions results in impaired performance on this multifaceted task, consistent with both structural (Pa et al., 2010) and functional neuroimaging (Moll et al., 2002; Zakzanis et al., 2005; Jacobson et al., 2011) studies of typical and atypical populations, which are described in greater detail below.

Three fMRI studies conducted with healthy adults utilizing either a verbal adaptation of the TMT or a version with an MRI-safe stylus implicated the frontal lobes when comparing Trails B vs. A performance. Two of these studies (Moll et al., 2002; Zakzanis et al., 2005) specifically implicated the left dorsolateral prefrontal cortex, while the third study implicated the right inferior and middle frontal gyri (Jacobson et al., 2011) along with the right precentral gyrus. All of these studies also noted the involvement of posterior brain regions while completing the TMT (and in particular when the B vs. A conditions were compared). Moll et al. (2002) noted the involvement of the intraparietal sulcus bilaterally. Zakzanis et al. (2005) reported left middle and superior temporal gyri activation and right cingulate and paracentral lobule activity. Finally, Jacobson et al. (2011) reported involvement of the left middle temporal and angular gyri.

Thus, there appears to be support from structural imaging studies of typical and atypical adults, lesion studies, and functional imaging for the importance of both the frontal lobes and posterior brain regions in the completion of the TMT. These findings fit with current thinking that different cognitive abilities are likely to be better understood from a functional (or structural) network perspective (for a review, see Park and Friston, 2013). Rather than focus on one modular region of the brain, network approaches suggest that it is the functioning of different clusters of brain regions that is important for higher-level cognition. Across studies, a number of different functional brain networks have been described, including the frontoparietal control, dorsal and ventral attention, somatosensory-motor, visual, language, and default mode networks (for a review, see Lee et al., 2012).

In an effort to add to this literature, the current study investigated how individual differences in structural covariance relate to TMT performance. Structural covariance refers to the observation that '… inter-individual differences in the structure of a brain region often covary with inter-individual differences in other brain regions (Alexander-Bloch et al., 2013a, p. 322). Our group has examined structural covariance using different methods, including graph analytic techniques (Alexander-Bloch et al., 2013b) and a method developed by Lerch et al. (2006) referred to as MACACC or Mapping Anatomical Correlations Across Cerebral Cortex. Using the latter technique, Lerch et al. demonstrated that cortical thickness correlation maps between a seed region in Broca's area and the rest of the cortex closely resembled white matter tractography maps generated from diffusion tensor imaging investigations. These findings suggested that correlations among regional gray matter measurements may indeed reflect the underlying white matter connectivity (and network structure of regions that are anatomically connected). Thus this technique is quite analogous to functional MRI, which relies on examining correlations among BOLD activation foci as a measure of functional connectivity. The MACACC technique has also identified structural covariance among regions implicated in highly replicated functional imaging networks, such as the default mode (Raznahan et al., 2011) and language (Lee et al., 2013) networks.

Furthermore, structural covariance has been found to be predictive of cognitive function (Lerch et al., 2006) and disease states (He et al., 2008). With regard to the former, Lerch and colleagues provided the first evidence that correlations among regional cortical thickness measurements index individual differences in intellectual abilities in typical youth. Following up on this, we investigated how individual differences in cortical thickness covariance related to vocabulary aptitude (Lee et al., 2013). Similar to Lerch's findings for intellectual abilities, we found that greater cortical thickness covariance among semantic hubs in the brain was related to higher scores on the Wechsler Vocabulary subtest (Lee et al., 2013).

In the current paper, we have chosen to examine cortical thickness covariance over the covariance of other measures of brain morphometry, such as regional surface area or gyrification, because prior work in our laboratory has demonstrated that individual differences in cortical thickness relate to variation in intellectual abilities (Shaw et al., 2006) as well as subclinical autistic and antisocial traits (Wallace et al., 2012). Thus, we applied a similar approach to the one used in Lee et al. (2013) to examine structural brain networks underpinning individual differences in TMT to test the hypothesis that stronger TMT performance will be associated with greater cross-cortical covariance in regions of cortex thought to be relevant to executive function abilities (e.g., the prefrontal cortex).

## Materials and methods

### Participants

The study's cross-sectional sample included 146 unrelated, typically-developing youth, ages 9–14 years, participating in an ongoing brain imaging study of single- and twin-birth children and adolescents being conducted in the Child Psychiatry Branch of the National Institute of Mental Health (NIMH; Giedd et al., 2009). The vast majority of participants were Caucasian (*n* = 121; 83%) and right-handed (*n* = 128; 88%). Data regarding age, IQ, and Trails Performance can be found in Table 1.

**Table 1:** **Demographic information about the sample and mean TMT age-adjusted Z-scores**.

	***Age***	**IQ**	**Trails B (s)**	**Trails B Age Z-score**	**Trails B–A (s)**	**Trails B–A Age Z-score**
*M*	12.23	114.28	28.50	−0.06	14.92	0.00
*SD*	1.80	12.08	10.66	0.91	9.08	1.00
Range	9–14	86–147	10–61	−1.76–2.82	0–48	−1.93–3.82

Inclusion criteria were as follows. Participants were required to: (a) be free of any developmental, learning, or psychiatric disorders as well any condition known to affect gross brain development; and (b) have provided useable data on both the TMT and a structural MRI scan (acquired on a GE 1.5 T scanner) that were acquired with 3 months of each other. [The vast majority (~98%) of participants completed testing and scanning within the same week].

Verbal or written assent was obtained from minors along with written consent from the parents. The NIMH Institutional Review Board approved the protocol.

### Cognitive measures

#### Wechsler intelligence scales

The Wechsler Abbreviated Scale of Intelligence (WASI) was administered to all participants (Wechsler, 1999) as an estimate of overall intellectual abilities.

#### Trail making test

All participants completed the Intermediate form of the Halstead-Reitan TMT (Reitan and Wolfson, 1993). As stated earlier, participants are asked to draw lines between encircled numbers (Part A) or to alternate between connecting encircled numbers and letters arranged on a page (Part B) as quickly as they can. Because the focus of the current study was on relations between individual differences in performance and anatomical coupling, scores on the different TMT measures were age-standardized by regressing the effects of age out of raw scores (i.e., the time to complete Trails B or the difference in time between Trails B and Trails A) and saving the standardized residuals (*M* = 0; *SD* = 1). The two primary variables considered in the current study were the age-regressed standardized residuals of Trails B Time and the Difference between Trails B and Trails A Completion Time (Trails B–A). Note that lower Z-scores denote better (faster) performance.

Prior to conducting primary analyses, data were inspected for normality and outliers. Of the 153 eligible participants with both a useable scan and TMT data, seven were excluded due to being outliers (>3 *SD* from the mean) on Trails A, Trails B or the difference between Trails B and A. This resulted in the current sample of 146 participants.

### MRI scan acquisition and processing methods

All MRI scans were acquired using the same General Electric 1.5 Tesla Signa Scanner at the National Institutes of Health Clinical Center in Bethesda, Maryland. Each participant contributed one scan. A three-dimensional spoiled gradient recalled echo sequence in the steady state, designed to optimize distinctions between gray matter, white matter, and cerebrospinal fluid was used to acquire 124 contiguous, 1.5-mm thick slices in the axial plane (*TE*/*TR* = 5/24 ms; flip angle = 45 degrees, matrix = 256 × 192, NEX = 1, FOV = 24 cm, acquisition time 9.9 min).

Montreal Neurological Institute's (MNI) automated CIVET pipeline was used for tissue classification and subsequent cortical thickness measurements. The native MRI scans were registered into standardized stereotaxic space and were corrected for non-uniformity artifacts (Sled et al., 1998) using a linear transformation (Collins et al., 1994). Tissue was classified into gray or white matter, spinal fluid, or background with a neural net classifier (Zijdenbos et al., 2002). Subsequently, the inner (white matter) and outer (pial) cortical surfaces were extracted using deformable surface-mesh models (MacDonald et al., 2000; Kim et al., 2005), and they were aligned non-linearly toward a standard template surface (Robbins et al., 2004).

Cortical thickness was quantified by measuring the linked distance between the white and pial surfaces (t-link metric) in native space (MacDonald et al., 2000; Lerch and Evans, 2005). A 30-mm surface-based diffusion-smoothing kernel (Chung et al., 2003) was utilized. These methods have been validated several ways. Validation methods include (a) manual measurements (Kabani et al., 2001), (b) population simulation (Lerch and Evans, 2005), and (c) validation within an Alzheimer's disease sample (Lerch et al., 2005).

All scans passed a two-stage quality assessment process which ensured the absence of (a) visible motion artifacts extending into the brain parenchyma in native images, and (b) visible errors in definition of the cortical ribbon based on an inspection of 3D reconstructions for the gray-white and pial surfaces in each scan. Furthermore, we graphically inspected the distribution of individual cortical thickness estimates within our sample at statistically significant peak foci to screen for outlier effects, as well as quantitatively tested for the lack of distorting outlier effects by re-running analyses after exclusion of any data point with a Cook's distance value of greater than 0.03. This value was calculated using the following formula: *d* = 4/*n* − *k* − 1 where *n* is the number of cases and *k* is the number of independent variables.

### Statistical analyses

The method we employ for analysis of structural covariance requires regressing the effects of age and sex out of vertex-level cortical thickness measurements to prevent observed anatomical coupling being confounded by the effects of age and sex on separate brain regions (Lerch et al., 2006). Age terms that were removed from cortical thickness measurements included age and age-squared, consistent with the findings from our laboratory on the longitudinal trajectory of cortical gray matter development from childhood to young adulthood (Giedd et al., 1999).

In order to evaluate if children with better scores on the TMT demonstrate a greater degree of structural covariance (particularly in regions such as the prefrontal cortex), an estimate of the relatedness of cross-cortical vertex-based thickness was needed. Analysis of vertex-wise cortical thickness correlations with overall mean cortical thickness (MCT) provides a computationally efficient alternative to calculating and then summarizing all possible vertex-vertex correlations in the brain (Lerch et al., 2006). Therefore, in keeping with prior work (Raznahan et al., 2011; Lee et al., 2013), we examine vertex-MCT coupling as a proxy for the relatedness of each vertex with all other vertices. This approach permits examination of the interaction between MCT and TMT performance continuously using regression in the complete sample of 146 participants rather than requiring participants to be categorized into arbitrary categories of high vs. low performance.

For primary analyses, regression was used to predict vertex thickness at 40,962 points in each hemisphere using a package written for use in R statistics developed by colleagues at MNI. In particular, we sought to determine if the relationship between MCT and the thickness of a particular vertex varied as a function of TMT performance. Thus, we were most interested in identifying vertices in which there was an interaction between MCT and TMT performance. Regression equations to test for this interaction were as follows:

*Trails B Time*:

Cortical thickness_(vertex j)_ = Intercept + ß1(MCT^1^) + ß2 (Trails B time^2^) + ß3(MCT^1*^ Trails B time^2^).

*Difference in time for Trails B vs. Trails A*:

Cortical thickness_(vertex j)_ = Intercept + ß1(MCT^1^) + ß2 (Trails B–A time^2^) + ß3(MCT^1*^ Trails B–A time^2^).

A False Discovery Rate (FDR) adjustment (Benjamini and Hochberg, 1995) was applied to control for multiple comparisons (i.e., 40,962 regression analyses per hemisphere). Specifically, FDR-adjusted q-values were generated for all terms in the regression equation—that is, the main effect of MCT, main effect of Trails, and the MCT^*^TMT performance interaction. The FDR threshold applied was *q* < 0.05.

#### Exploratory age group analyses

Lastly, given that the focus of this special issue is on the *development* of executive functions in childhood, we ran exploratory analyses in order to begin to investigate if TMT-coupling relations vary as a function of age in childhood. We did this in two ways. First, we ran a linear regression predicting vertex-level cortical thickness using the following dependent variables: MCT (age-standardized), TMT performance (age-standardized), age group (above or below the median age of 12.48) and their interactions (both two-way interactions and the three-way interaction). Regression equations used for these analyses are as follows.

*Trails B Time*:

Cortical thickness_(vertex j)_ = Intercept + ß1(MCT^1^) + ß2 (Trails B time^2^) + ß3 (Age Subgroup) + ß4 (MCT^1*^ Trails B time^2^) + ß5 (MCT^1*^Age Subgroup) + ß6 (Trails B time^2*^Age Subgroup) + ß7 (MCT^1*^ Trails B time^2*^Age Subgroup).

Difference in time for Trails B vs. Trails A:

Cortical thickness_(vertex j)_ = Intercept + ß1(MCT^1^) + ß2 (Trails B–A time^2^) + ß3 (Age Subgroup) + ß4 (MCT^1*^ Trails B–A time^2^) + ß5 (MCT^1*^Age Subgroup) + ß6 (Trails B–A time^2*^Age Subgroup) + ß7 (MCT^1*^ Trails B–A time^2*^Age Subgroup).

For these analyses, we were most interested in the three-way interaction for MCT^*^TMT^*^Age subgroup, as a significant interaction would suggest that the relations between anatomical coupling within the context of TMT performance varied as a function of age.

Second, we divided the sample into younger and older participants by splitting the group at the median age. We then re-ran the primary regression analyses in the younger and older samples to qualitatively compare the findings. This will be described in greater detail in the Results section.

## Results

In this manuscript, our primary research question was as follows: Is stronger TMT performance in childhood (as measured by time to complete Trails B and the difference between Trails B and A) associated with greater cross-cortical covariance in regions of cortex thought to be relevant to executive functions (e.g., the prefrontal cortex)? Stated another way, is the thickness of the prefrontal cortex and other cortical regions more highly correlated with the thickness of the rest of cortex (as estimated by MCT) in those with higher TMT scores?

This question was evaluated separately at every vertex in each hemisphere in the complete sample of 146 participants using the regression equations described above in the Materials and Methods section. In particular, we were interested in whether the MCT^*^TMT performance interaction was significant, as this would indicate that the strength of the relationship between MCT and a particular vertex's thickness varied as a function of TMT performance.

**Figure 1:**
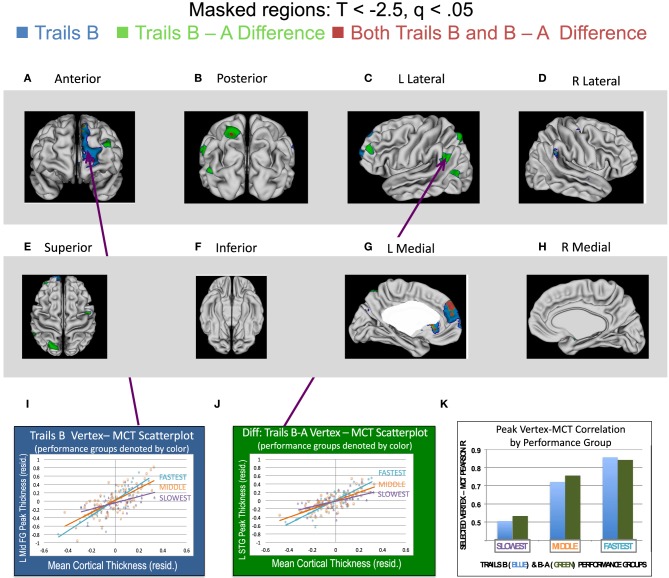
**Regions associated with greater cross-cortical coupling for those with stronger performance on Trails B, the Differences between Trails B and A, and Both Trails B and the B–A Difference**. Two sets of linear regression analyses predicting cortical thickness at each vertex in both hemispheres were run in the complete sample (*n* = 146) of participants in order to evaluate if coupling between mean cortical thickness (MCT) and vertex thickness varied as a function of individual differences in either (1) Trails B time (age-adjusted) or (2) the difference in time between Trails B and A (age-adjusted). The regression equations were as follows. (1) For Trails B time: Cortical thickness _(vertex j)_ = Intercept + ß_1_(MCT) + ß_2_(Trails B time) + ß_3_(MCT^*^Trails B). (2) For the Difference in time for Trails B vs. Trails A: Cortical thickness _(vertex j)_ = Intercept + ß_1_(MCT^1^) + ß_2_(Trails B–A time) + ß_3_(MCT^*^Trails B - A time). Note that for these analyses, the vertex-level dependent variables and MCT were age and sex standardized. (See Materials and Methods for details). The Trails B and B–A variables were age-standardized. *T*-statistics associated with the MCT^*^Trails interaction were corrected for multiple comparisons using a False Discovery Rate adjustment. Only those vertices with *T*s < −2.5 and *q*s < 0.05 are displayed in this figure in **(A–H)**. (Note that *t*-values are negative, because faster or shorter times are indicative of better performance). In these panels, blue vertices are those in which the MCT^*^Trails B interaction was significant, such that tighter coupling between MCT and the thickness of that vertex was found for those who were faster (better) on Trails B. Green vertices are those in which the MCT^*^Trails B vs. A Difference score interaction was significant, such that stronger coupling was found for those with better performance (i.e., smaller differences in time between Trails B and A). Vertices in red are those for which both of the regression equations' interaction terms were significant. **(I,J)** display relations between MCT and a vertex in the middle frontal gyrus (MNI coordinates = *x* = −8, *y* = 68, *z* = 3) or the middle temporal gyrus (MNI coordinates: *x* = −47, *y* = −70, *z* = 16), respectively, for performers stratified into three groups: the best/fastest performers shown in turquoise (those with scores in the lower quartile—denoting faster performance; *n* = 37), the middle performers in orange (middle 50% of sample; *n* = 72) and worst/slowest performers in purple (those with scores in the upper quartile—denoting slower performance; *n* = 37). As can be seen, a steeper regression line was associated with better performance. (Please note that performance was stratified into the three groups for illustrative purposes only here. The regression equations included a continuous measurement of performance on the TMT within the complete sample of 146 participants.) Lastly, **(K)** illustrates the Pearson *r* correlation coefficient values for (1) MCT and the selected vertex for Trails B and (2) MCT and the selected vertex for the difference between Trails B and A for the three subgroups included in the scatterplots shown in **(I,J)**. These values are shown for Trails B performance with the blue bars and the Difference between Trails B–A performance with the green bars. As can be seen, as performance group moves from slowest to fastest, the correlation between the pictured vertex and MCT increases.

Regions in which statistically significant interactions were found between MCT and either Trails B Time (age-adjusted) or the Difference between Trails B and A (age-adjusted) are presented in Figure 1. Blue vertices are those in which the MCT^*^Trails B interaction was significant (following FDR correction, *q* < 0.05 for all terms in the regression equation), such that tighter correlations between MCT and the thickness of that vertex were found for those who were faster (better) on Trails B. Green vertices are those in which the MCT^*^Trails B vs. A Difference score interaction was significant, such that stronger coupling was found for those with better performance (i.e., smaller differences in time between Trails B and A). Vertices in red are those for which both of the regression equations' interaction terms were significant. Because the focus of the manuscript was on regions of the cortex in which MCT and vertex thickness correlations varied as a function of TMT performance, we have elected to leave the findings for main effects of MCT and TMT performance out of Figure 1. However, this information has been included in Supplementary Figures S1 and S2 for Trails B and Trails B–A, respectively.

As can be seen in Figure 1, a large swath of cortex in the superior and medial prefrontal cortex was more tightly coupled with the rest of the cortical ribbon in those who were faster at Trails B. When the difference between Trails B and A was considered, several smaller clusters of vertices were found to be more tightly coupled with the thickness of the rest of the cortex in better performers, including an overlapping region in the medial prefrontal cortex associated with better Trails B performance described above (in red in the figure). Additional regions included a cluster of vertices in dorsolateral prefrontal cortex, two small clusters near the temporal-parietal junction, and a cluster of vertices in superior parietal lobule (including a small region that overlapped with Trails B performance as shown in red). Lastly, there were also a few regions in which tighter coupling between MCT and vertex thickness was associated with poorer TMT performance. These results are summarized in Supplementary Figure S3.

To complement these analyses and demonstrate that the clusters of vertices displayed in Figure 1 were associated with a greater degree of coupling with the rest of the cortex in those who were better performers (based on age-adjusted scores), we dichotomized the complete sample of 146 participants into those with scores in the lower and upper quartiles of the sample based on their age-adjusted Trails B score (or the difference in time between Trails B and A–age-adjusted). We then ran correlations between the thickness of the peak vertex identified in prior analyses and all vertices in the left and right hemisphere in the two groups—high/fast performers (those with scores in the lower quartile—denoting faster performance) and low/slow performers (those with scores in the upper quartile—denoting slower performance).

**Figure 2:**
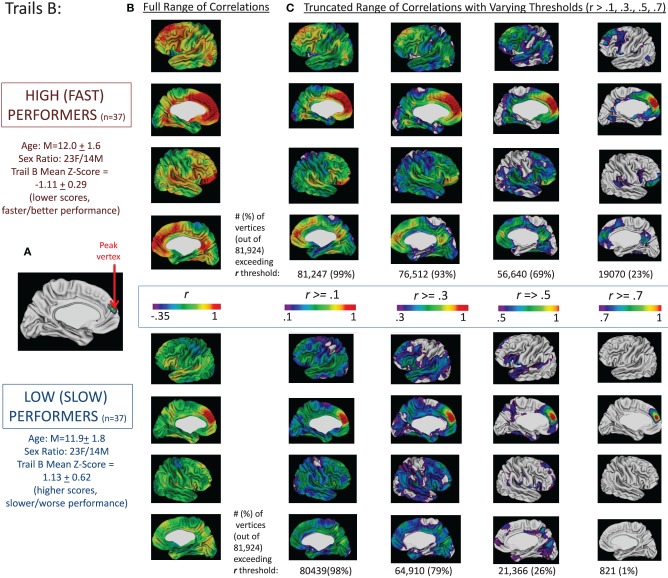
**Correlations between peak vertex thickness in the medial prefrontal cortex and the rest of the cortex in high (*n* = 37) and low (*n* = 37) performers on Trails B**. The complete sample of 146 participants was divided into quartiles based on performance on Trails B (age-adjusted scores). We then ran two sets of correlations between the thickness of the peak vertex identified in prior analyses in medial prefrontal cortex [shown in **(A)**; MNI coordinates: *x* = −9, *y* = 51, *z* = 14] and all vertices in the left and right hemisphere for the group of high/fast performers (those with scores in the lower quartile—denoting faster performance; *n* = 37) and the low/slow performers (those with scores in the upper quartile—denoting slower performance; *n* = 37). The resulting correlation coefficients were projected onto the cortical sheet and are presented in **(B)**. In order to illustrate differences in the number of vertices that exceeded different correlation coefficient thresholds, the correlation range evaluated was truncated and **(C)** presents the regions (and number of vertices) in which the correlation coefficients exceeded the following thresholds: *r* > 0.1, 0.3, 0.5, and 0.7. These values (i.e., number of vertices falling above the different thresholds) are provided under the four images of the brains associated with each threshold for the high and low groups.

**Figure 3:**
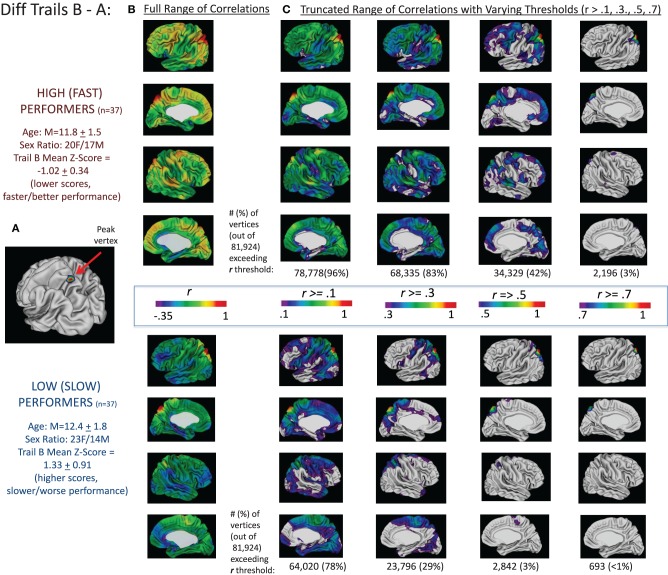
**Correlations between peak vertex thickness in the superior parietal lobule and the rest of the cortex in high (*n* = 37) and low (*n* = 37) performers on the Trails B–A difference score**. Analogous to the procedures described in Figure 2, the complete sample of 146 participants was divided into quartiles based on performance on the difference in Trails B and A times (age-adjusted scores). We then ran two sets of correlations between the thickness of the peak vertex identified in prior analyses in superior parietal lobule [shown in **(A)**; MNI coordinates: *x* = −22, *y* = −68, *z* = 62] and all vertices in the left and right hemisphere for the group of high/fast performers (those with scores in the lower quartile—denoting faster performance; *n* = 37) and the low/slow performers (those with scores in the upper quartile—denoting slower performance; *n* = 37). The resulting correlation coefficients were projected onto the cortical sheet and are presented in **(B)**. In order to illustrate differences in the number of vertices that exceeded different correlation coefficient thresholds, the correlation range evaluated was truncated and **(C)** presents the regions (and number of vertices) in which the correlation coefficients exceeded the following thresholds: *r* > 0.1, 0.3, 0.5, and 0.7. These values (i.e., number of vertices falling above the different thresholds) are provided under the four images of the brains associated with each threshold for the high and low groups.

These findings are summarized for Trails B in Figure 2 and for the difference between Trails B and A in Figure 3. For Trails B, the vertex in which the highest *t*-value was found for the interaction between MCT and TMT performance—referred to as the “peak vertex”—was identified in the medial prefrontal cortex (see Figure 2A). The thickness of this vertex was correlated with all other vertices in the high/fast and low/slow performing groups separately. The resulting correlation coefficients were projected onto the cortex and are presented in Figure 2B. In order to illustrate differences in the number of vertices that exceeded different correlation coefficient thresholds, the correlation range evaluated was truncated and Figure 2C presents the regions (and number of vertices) in which the correlation coefficients exceeded the following thresholds: *r* > 0.1, 0.3, 0.5, and 0.7. These values (i.e., number of vertices falling above and below the different thresholds) were compared for high/fast and low/slow performers utilizing chi-square. For all comparisons, the chi-square results were significant (all χ^2^s > 100, *p*s < 0.001) in favor of the higher/faster performers having a greater proportion of vertices that exceeded the stated correlation coefficient threshold. Analogously, for the difference between Trails B–A, the peak in the superior parietal lobule is used as the seed and the corresponding correlations are presented in Figures 2B,C. Lastly, for Trails B–A, correlation coefficient maps for the peak vertices in the middle and superior temporal lobe clusters and the dorsal and medial prefrontal cortex clusters are provided in Supplementary Figure S4.

### Exploratory analyses examining the effects of age on TMT-anatomical coupling findings

Given that the focus of the current special issue is on the development of executive functions, we undertook several exploratory analyses to evaluate differential age-effects on the TMT performance-coupling findings described above for the complete sample. Specifically, we ran a linear regression predicting vertex-level cortical thickness using the following dependent variables: MCT (age-standardized), TMT performance (age-standardized), age group (above or below the median age of 12.48) and their interactions (both two-way interactions and the three-way interaction). See the end of the Materials and Methods section for the equations utilized for Trails B and the difference between Trails B and A. For these analyses, we were most interested in the three-way MCT^*^TMT^*^Age group interaction, as this would suggest that the relations between anatomical coupling within the context of TMT performance varied as a function of age.

For Trails B, only three small regions were predicted significantly by the three-way interaction (*q* < 0.05). These included small regions in inferior medial prefrontal cortex, inferior somatosensory cortex, and the posterior cingulate. The approximate locations of these three regions are identified in Supplementary Figure S5 with asterisks.

For these three-way interactions, results were such that tighter coupling was associated with better performance on Trails B (age-standardized) in the younger but not older subgroup. (For the older subgroup, the general trend in the data was for tighter coupling in the three regions being associated with *poorer* performance).

For the difference between Trails B and A, no statistically significant three-way interactions were identified, suggesting that the coupling-TMT performance findings were not modified by age. In addition to evaluating the occurrence of three-way interactions for Trails B and the difference between Trails B and A, we also divided our sample into two age-based subgroups (Younger: age less than the group median of 12.48; Older: age greater than or equal to the group median) in order to examine age effects in a more qualitative fashion. We then re-ran the initial regression equation used to answer the main study questions in these two subgroups: vertex thickness ~ MCT + TMT + MCT^*^TMT performance. The MCT^*^TMT performance interaction results for the age-adjusted Trails B findings and the age-adjusted Trails B–A findings were projected onto the cortical surface in Supplementary Figures S5, S6, respectively. Cooler colors in these figures represent those in which the MCT^*^TMT performance interaction was significant in the whole sample, the younger subgroup, or both. In contrast, the warm colors represent regions in which the MCT^*^TMT interaction was significant for the older subgroup or both the older subgroup and the whole sample.

The results of the three-way MCT^*^TMT^*^Age interaction and the subgroup analyses suggest that age-effects on TMT-coupling are small within this limited age-range. However, these small effects suggest that younger age is associated with coupling among a greater number of cortical regions. This was tested by comparing the number of vertices that exceeded the FDR-corrected threshold (*q* < 0.05) for the MCT^*^TMT interaction for the younger and older subgroups. For both Trails B and the difference between Trials B and A, the chi-square findings were highly significant. For Trails B, 1229 vertices exceeded the threshold in the younger subgroup while only 297 exceeded this threshold in the older subgroup [χ^2(1)^ = 573, *p* < 0.001]. Similarly, for the B–A difference, 800 vertices exceeded the threshold for the younger subgroup compared to 256 in the older subgroup [χ^2(1)^ = 281, *p* < 0.001].

## Discussion

Adding to the literature on the neural correlates of executive function in childhood, here we demonstrate that individual differences on a commonly-administered executive function task, the Halstead-Reitan TMT, relate to the degree of anatomical coupling between the left prefrontal cortex and other distributed cortical regions. In particular, we found that for youth who were faster than their peers on Trails B (age-adjusted scores), there was greater coupling between a large swath of the prefrontal cortex, including portions of Brodmann areas 9 (dorsolateral prefrontal cortex) through 11 and the anterior cingulate, and the rest of cortex. When the difference between Trails B and A (age-adjusted) was considered, a network of mostly left-lateralized regions was found to be more strongly coupled with the rest of cortex, including clusters of vertices in the dorsolateral and dorsomedial prefrontal cortex, the posterior middle and superior temporal gyri (corresponding roughly to the angular and supramarginal gyri, respectively), and the superior parietal lobule.

These findings are the first to demonstrate how individual differences in structural (as opposed to functional) covariance relate to performance differences in executive functioning, a group of higher-level cognitive abilities that are believed to be important for academic outcomes (Blair and Razza, 2007) and are impaired in numerous developmental disorders (Ozonoff and Jensen, 1999). Despite the current study's focus on structural covariance, these findings are remarkably consistent with fMRI investigations into the functional correlates of TMT performance. Specifically, two studies (Moll et al., 2002; Zakzanis et al., 2005) implicated the left dorsolateral prefrontal cortex when Trails B performance was contrasted with Trails A. Furthermore, these two studies and a study conducted by Jacobson et al. (2011) reported the involvement of several posterior brain regions when Trails B activation was contrasted with Trails A activation. These included the intraparietal sulcus bilaterally (analogous to our supramarginal and angular gyri findings; Moll et al., 2002), the left middle and superior temporal gyri (Zakzanis et al., 2005; Jacobson et al., 2011), the angular gyrus (Jacobson et al., 2011), and the superior parietal lobule (for Trails B performance in particular; Allen et al., 2011).

Taken together, our structural covariance findings in concert with existing fMRI data provide support that a network of frontal and posterior brain regions is involved with successful TMT performance. Our study importantly extends the existing literature to include children for whom executive functioning abilities are developing. In addition, the current study's findings converge with a recent meta-analysis of adult lesion studies that strongly demonstrated that damage to brain regions other than the frontal lobes was just as likely to impair Trails B (and other executive test) performance as damage to the frontal lobes. Given the complexity of executive function tasks and the number of lower-level cognitive abilities that are involved (e.g., basic visual perception, focused attention, motor coordination and speed), it is not surprising that a network of regions working in unison is likely to underlie successful performance both in adulthood and childhood.

Analogous to our finding of greater structural covariance between the dorsolateral prefrontal cortex and the rest of the cortical ribbon, Cole and colleagues reported higher degrees of global functional connectivity in the lateral prefrontal cortex in individuals with higher scores on measures of cognitive control (such as classic fluid intelligence tests; Cole et al., 2012). Moreover, an examination of the regions implicated in the current investigation of the TMT reveals an overlap with regions in the frontoparietal control and the default mode networks, two networks first described in functional connectivity studies (for a review, see Lee et al., 2012). In fact, it has been suggested that these networks are two of the most functionally connected in the brain (Cole et al., 2010). Thus, it is not surprising that higher degrees of structural covariance in these regions relates to higher performance on a complex, multifaceted executive function task, such as the TMT.

With regard to the examination of the impact of age on our TMT-coupling results, we found a small effect on these relations. However, the trend in our data tentatively suggested that anatomical coupling across *multiple* regions may be of greater importance for TMT success in younger participants, during a developmental period when executive function abilities are rapidly developing. In contrast, as children and adolescents age, anatomical coupling in multiple regions may be less crucial for better performance. Instead, it could be that with age comes some regional specialization and greater reliance on cross-cortical coupling of a few select regions (e.g., reliance on the coupling of the dorsolateral prefrontal cortex in particular).

Given the importance of the prefrontal cortex in the current study and others examining executive functioning using different methodologies, we would be remiss if we did not focus some of our discussion on the importance of the frontal lobes to executive abilities in particular. In a review paper from 2001, Miller and Cohen provided an integrative theory about the functioning of the prefrontal cortex (Miller and Cohen, 2001). Based on a synthesis of neuroimaging, neurophysiological, anatomical, and computational investigations, they likened the prefrontal cortex to a “switch operator” in a rail system. Using this metaphor, they described the activity of the prefrontal cortex as a map that delineates which “tracks” or neural pathways are necessary for the completion of different cognitive tasks.

In this review, Miller and Cohen (2001) discussed the importance of the prefrontal cortex in maintaining “active representations” necessary to complete novel tasks requiring goal-directed behavior and flexibility. They suggested that one of the aspects of the prefrontal cortex that makes it unique is its ability to maintain active representations in the face of interference. Another unique feature of the prefrontal cortex is its high level of interconnectivity with sensory, motor, and limbic systems within the brain. These two qualities, among others, make the prefrontal cortex ideally-suited to serve as a “hub” and coordination center for higher-level cognitive abilities that require the work of multiple neuroanatomic regions.

In line with Miller and Cohen's conceptualization, more recent accounts of prefrontal cortex functioning such as the “gateway hypothesis” (Burgess et al., 2007) describe the rostral prefrontal cortex (roughly Brodmann area 10), an area implicated in the current investigation, as a “supervisory attentional gateway” that permits “stimulus-oriented” or “stimulus-independent” focused attention. These authors argue that the lateral rostral prefrontal cortex is more associated with the former, while the medial rostral prefrontal cortex is more associated with the latter. In the current investigation, both the lateral and medial prefrontal cortex were found to be more coupled in youth with higher TMT performance. Greater coupling in both of these regions certainly fits with TMT task demands—that is, one must attend to external stimuli (the encircled symbols on the page) and internal representations (maintaining a rote sequence of letters and numbers) in order to perform successfully on the task.

The current findings are also in line with the WHACH (what-how, abstract, cold-hot) model of prefrontal cortex functioning (O'Reilly, 2010). This model differentiates dorsal and ventral prefrontal functioning and suggests that the dorsal pathway is associated with guiding “how” to cope with information (i.e., “… transforming perception into action,” p. 355) while the ventral pathway is associated with identifying “what” semantic information is relevant for a particular task (i.e., “… guiding the selection and retrieval of semantic/linguistic knowledge,” p. 336). O'Reilly points out that the dorsal portions of the prefrontal cortex appear to be particularly relevant for transforming sensory inputs into motor outputs and for sequential ordering. These are two key aspects of successful TMT performance. Thus, higher coupling of the dorsal prefrontal cortex in better TMT performers provides additional support for the “how” conceptualization of dorsal prefrontal functioning.

Given the current study's findings and those of others, it may be that the prefrontal cortex represents a hub for higher-level executive abilities due to its inclusion in highly interconnected networks (dorsal portion of the frontoparietal control network and dorsal-medial portion of the default mode network). Based on the work of Buckner et al. (2009), it appears that all of the regions that were found to be more highly coupled in those with better TMT performance may indeed be locations of cortical hubs. Why might the “hub” regions implicated here be more coupled with the rest of cortex in youth who perform better on the TMT task? One possible explanation draws upon the Hebbian learning notion that neurons that “fire together, wire together” (Hebb, 1949). Thus, it may be that in youth who are better at executive tasks, the coordinated use of different regions of the brain, including the prefrontal cortex, results in a higher degree of anatomical coupling among these regions. An alternate explanation is that genetic factors contributing to the development of these brain regions are shared, and that youth who are better at these tasks are predisposed to more coordinated development in these regions.

The cross-sectional nature of this investigation precludes drawing any conclusions about these alternatives, a limitation of our study design. Another limitation of our study is that we focused on just one executive task, thus reducing the generalizability of our findings to other executive abilities. Furthermore, given the small age range of the sample studied here (9–14 years), we were only able to examine age-TMT-structural covariance relations in a preliminary way. A rigorous examination of age by performance effects on anatomical coupling, particularly within the context of a longitudinal study design, will be a crucial next step in understanding the complex unfolding of the development of executive abilities and how individual differences in performance emerge over time.

Despite these limitations, this study is the first of its kind to highlight the importance of structural covariance in relation to individual differences in executive function abilities in youth. Thus, it adds to the growing literature on the neural correlates of childhood executive functions and identifies neuroanatomic coupling as a biological substrate that may contribute to typical and atypical executive development. Consistent with fMRI connectivity work (Lee et al., 2012; Park and Friston, 2013), the present study demonstrates that successful performance on a multiply-determined executive function task is associated with greater anatomical coupling between the prefrontal cortex and other broadly-distributed cortical regions during childhood and adolescence. Thus, this study of individual differences in the context of typical development suggests that disorders of childhood associated with executive dysfunction (i.e., lower scores on tasks like the TMT), such as attention deficit hyperactivity disorder and autism spectrum disorder, might demonstrate more localized anatomical coupling in the frontal lobe and other regions.

## Author contributions

Nancy Raitano Lee, Gregory L. Wallace, and Jay N. Giedd contributed to study design. Liv S. Clasen prepared data for analysis. Nancy Raitano Lee analyzed data and wrote the manuscript. Gregory L. Wallace, Liv S. Clasen, Armin Raznahan, and Jay N. Giedd critically revised the manuscript.

### Conflict of interest statement

The authors declare that the research was conducted in the absence of any commercial or financial relationships that could be construed as a potential conflict of interest.

## Footnotes

^1^The MCT measure utilized here was residualized, with the variance associated with age, age^2^, and sex removed.

^2^The TMT measures utilized here (Trails B and the difference between Trails B and A) were residualized, with the variance associated age removed.

## Supplementary material

Supplementary Figure S1 **Regions in which there were main effects of mean cortical thickness, Trails B performance, and their interaction in predicting vertex thickness**. This figure supplements Figure 1 in the main document. Linear regression analyses predicting cortical thickness at each vertex in both hemispheres were run in the complete sample (*n* = 146) of participants in order to evaluate the effects of mean cortical thickness, Trails B age-adjusted scores, and their interaction. The regression equation was as follows: Cortical thickness _(vertex j)_ = Intercept + ß_1_(MCT) + ß_2_(Trails B time) + ß_3_(MCT^*^Trails B). Note that for these analyses, the vertex-level dependent variables and MCT were age and sex standardized. (See Materials and Methods for details). The Trails B variables were age-standardized. *T*-statistics associated with each of the effects in the regression equation were corrected for multiple comparisons using a False Discovery Rate adjustment. Only those vertices with *q*s < 0.05 (associated with a *T*-threshold of 2.5) are displayed in this figure in **(A–H)**. Vertices in purple are those in which a main effect of MCT was found; vertices in blue are those in which main effects of MCT and Trails B were found such that thinner cortex was associated with better performance; vertices in turquoise green are those in which main effects of MCT and Trails B were found such that thicker cortex was associated with better performance; lastly, vertices in yellow are those in which an MCT^*^Trails B interaction was found such that greater coupling was associated with better performance.

Supplementary Figure S2 **Regions in which there were main effects of mean cortical thickness, Trails B–A performance, and their interaction in predicting vertex thickness**. This figure also supplements Figure 1 in the main document. Linear regression analyses predicting cortical thickness at each vertex in both hemispheres were run in the complete sample (*n* = 146) of participants in order to evaluate the effects of mean cortical thickness, Trails B–A age-adjusted scores, and their interaction. The regression equation was as follows: Cortical thickness _(vertex j)_ = Intercept + ß_1_(MCT) + ß_2_(Trails B–A time-age-adjusted) + ß_3_(MCT^*^Trails B–A time- age-adjusted). Note that for these analyses, the vertex-level dependent variables and MCT were age and sex standardized. (See Materials and Methods for details). The Trails B–A variables were age-standardized. *T*-statistics associated with each of the effects in the regression equation were corrected for multiple comparisons using a False Discovery Rate adjustment. Only those vertices with *q*s < 0.05 (associated with a *T*-threshold of 2.5) are displayed in this figure in **(A–H)**. Vertices in purple are those in which a main effect of MCT was found; vertices in blue are those in which main effects of MCT and Trails B–A were found such that thinner cortex was associated with better performance; vertices in turquoise green are those in which main effects of MCT and Trails B–A were found such that thicker cortex was associated with better performance; lastly, vertices in yellow are those in which an MCT^*^Trails B–A interaction was found such that greater coupling was associated with better performance.

Supplementary Figure S3 **Regions associated with greater cross-cortical coupling for those with poorer performance on Trails B, the Differences between Trails B and A, and Both Trails B and the B–A Difference**. This figure complements Figure 1 in the main document in that it displays regions of the cortex in which greater coupling was associated with *poorer* performance. Two sets of linear regression analyses predicting cortical thickness at each vertex in both hemispheres were run in the complete sample (*n* = 146) of participants in order to evaluate if coupling between mean cortical thickness (MCT) and vertex thickness varied as a function of individual differences in either (1) Trails B time or (2) the difference in time between Trails B and A. The regression equations were as follows. (1) For Trails B time: Cortical thickness _(vertex j)_ = Intercept + ß_1_(MCT) + ß_2_(Trails B time) + ß_3_(MCT^*^Trails B). (2) For the Difference in time for Trails B vs. Trails A: Cortical thickness _(vertex j)_ = Intercept + ß_1_(MCT^1^) + ß_2_(Trails B–A time) + ß_3_(MCT^*^Trails B − A time). Note that for these analyses, the vertex-level dependent variables and MCT were age and sex standardized. (See Materials and Methods for details). The Trails B and B–A variables were age-standardized. *T*-statistics associated with the MCT^*^Trails interaction were corrected for multiple comparisons using a False Discovery Rate adjustment. Only those vertices with *T*s > 2.5 and *q*s < 0.05 are displayed in this figure in **(A–H)**. (Note that *t*-values are positive, because slower or longer times are indicative of poorer performance.) In these panels, blue vertices are those in which the MCT^*^Trails B interaction was significant, such that tighter coupling between MCT and the thickness of that vertex was found for those who were *slower* (worse) on Trails B. Green vertices are those in which the MCT^*^Trails B vs. A Difference score interaction was significant, such that stronger coupling was found for those with worse performance (i.e., greater differences in time between Trails B and A). Vertices in red are those for which both of the regression equations' interaction terms were significant. Lastly, **(I,J)** display relations between MCT and the thickness of two vertices in the inferior frontal gyrus—one associated with Trails B (MNI coordinates: *x* = 25, *y* = 28, *z* = −17) and the other associated with Trails B − A time (MNI coordinates: *x* = 20, *y* = 26, *z* = −20). Regression lines are for performers in three groups: the best/fastest performers shown in turquoise (those with scores in the lower quartile—denoting faster performance; *n* = 37), the middle performers in orange (middle 50% of sample; *n* = 72) and worst/slowest performers in purple (those with scores in the upper quartile—denoting slower performance; *n* = 37). As can be seen, a steeper regression line was associated with poorer performance. (Please note that performance was stratified into the three groups for illustrative purposes only here. The regression equations included a continuous measurement of performance on the TMT within the complete sample of 146 participants.)

Supplementary Figure S4 **Correlations between peak vertex thickness for clusters in the middle temporal, superior temporal, dorsolateral prefrontal, and dorsomedial prefrontal cortex in high (***n*** = **37**) and low (***n*** = **37**) performers on the Trails B–A difference score**. Analogous to the procedures described in Figure 3, the complete sample of 146 participants was divided into quartiles based on performance on the difference in Trails B and A times (age-adjusted scores). We then ran two sets of correlations between the thickness of the peak vertex identified in prior analyses in the **(A)** posterior middle temporal gyrus, **(B)** posterior superior temporal gyrus, **(C)** dorsolateral prefrontal cortex, and **(D)** dorsomedial prefrontal cortex and all vertices in the left and right hemisphere for the group of high/fast performers (those with scores in the lower quartile—denoting faster performance; *n* = 37) and the low/slow performers (those with scores in the upper quartile—denoting slower performance; *n* = 37). The resulting correlation coefficients were projected onto the cortical sheet and can be viewed in **(A)** through **(D)**. Note: MNI coordinates for peaks included in this figure were as follows: **(A)** posterior middle temporal gyrus: *x* = −47, *y* = −70, *z* = 16; **(B)** posterior superior temporal gyrus: *x* = −57, *y* = −54, *z* = 34; **(C)** dorsolateral prefrontal cortex: *x* = −39, *y* = 51, *z* = 16; **(D)** dorsomedial prefrontal cortex: *x* = −4, *y* = 53, *z* = 29.

Supplementary Figure S5 **Regions in which an interaction between mean cortical thickness Trails B performance were found for younger participants (*n* = 73), older participants (*n* = 73), and the whole sample (*n* = 146)**. In order to investigate whether TMT-coupling relations vary as a function of age in childhood, we divided the sample into younger and older participants by splitting the group at the median age (12.48). We then re-ran the primary regression analyses [Cortical thickness _(vertex j)_ = Intercept + ß_1_(MCT) + ß_2_(Trails B time-age-adjusted) + ß_3_(MCT^*^Trails B-age-adjusted)] in the younger (*n* = 73) and older (*n* = 73) subgroups to qualitatively compare the findings. Note that for these analyses, the vertex-level dependent variables and MCT were age and sex standardized. (See Materials and Methods for details). The Trails B variables were age-standardized. Vertices exceeding the FDR-adjusted threshold for MCT^*^Trails B interaction (*q* < 0.05; *T*-threshold of 2.5) were projected onto the cortex using the following color code. (1) Vertices associated with a statistically significant MCT^*^Trails B interaction in the whole sample were coded royal blue; (2) vertices that were only found to be statistically significant in the younger sample were coded turquoise blue; (3) vertices associated with a statistically significant interaction in analyses of both the whole sample and the younger sample were coded green; (4) vertices with statistically significant interactions in the older subgroup were coded orange; and (5) vertices with statistically significant interaction terms in both analyses of the older subgroup and the whole sample were coded red. In order to evaluate age-effects using a more rigorous technique statistically, we also ran a series of linear regression analyses predicting vertex-level cortical thickness using the following equation: Cortical thickness_(vertex j)_ = Intercept + ß1(MCT) + ß2(Trails B time) + ß3 (Age Subgroup) + ß4 (MCT^*^Trails B time-age adjusted) + ß5 (MCT^*^Age Subgroup) + ß6 (Trails B time-age adjusted ^*^Age Subgroup) + ß7 (MCT^*^Trails B time-age adjusted^*^Age Subgroup). Again note that for these analyses, the vertex-level dependent variables and MCT were age and sex standardized (see Materials and Methods for details) and the Trails B variables were age-standardized. Regions with an asterisk (^*^) denote the approximate location of the statistically significant three-way interactions that survived the FDR adjusted *T*-value of 2.7 (*q* < 0.05). For the three regions in which there was a three-way interaction, the findings were such that greater coupling was associated with better Trails B (age-adjusted) performance in younger participants. The general trend in the data was for the opposite to be true in the older subgroup—that is tighter coupling was associated with poorer performance.

Supplementary Figure S6 **Regions in which an interaction between mean cortical thickness Trails B–A performance were found for younger participants (*n* = 73), older participants (*n* = 73), and the whole sample (*n* = 146)**. In order to investigate whether TMT-coupling relations vary as a function of age in childhood, we divided the sample into younger and older participants by splitting the group at the median age (12.44). We then re-ran the primary regression analyses [Cortical thickness _(vertex j)_ = Intercept + ß_1_(MCT) + ß_2_(Trails B–A time-age-adjusted) + ß_3_(MCT^*^Trails B–A-age-adjusted)] in the younger (*n* = 73) and older (*n* = 73) subgroups to qualitatively compare the findings. Vertices with interaction terms exceeding the FDR-adjusted threshold for regression analyses (*T* > 2.5; *q* < 0.05) were projected onto the cortex using the following color code. (1) Vertices associated with statistically significant MCT^*^Trails B–A interaction terms in the whole sample were coded royal blue; (2) vertices that were only found to be statistically significant in the younger sample were coded turquoise blue; (3) vertices associated with a statistically significant interaction in analyses of both the whole sample and the younger sample were coded green; (4) vertices with statistically significant interactions in the older subgroup were coded orange; and (5) vertices with statistically significant interaction terms in both analyses of the older subgroup and the whole sample were coded red. In order to evaluate age-effects using a more rigorous technique statistically, we also ran a series of linear regression analyses predicting vertex-level cortical thickness using the following equation: Cortical thickness_(vertex j)_ = Intercept + ß1(MCT) + ß2(Trails B–A time) + ß3 (Age Subgroup) + ß4 (MCT^*^Trails B–A time-age-adjusted) + ß5 (MCT^*^Age Subgroup) + ß6 (Trails B–A time-age adjusted ^*^Age Subgroup) + ß7 (MCT^*^Trails B time-age adjusted^*^Age Subgroup). Again note that for these analyses, the vertex-level dependent variables and MCT were age and sex standardized (see Materials and Methods for details) and the Trails B variables were age-standardized. Unlike Trails B performance, no statistically significant three-way interactions were found (all *q*s > 0.05).
