# Supplementary figures and images for: Trail making test performance in youth varies as a function of anatomical coupling between the prefrontal cortex and distributed cortical regions
Source: Front Psychol. 2014 Jul 1;5:496. doi: 10.3389/fpsyg.2014.00496 (PMC4077145; doi:10.3389/fpsyg.2014.00496)

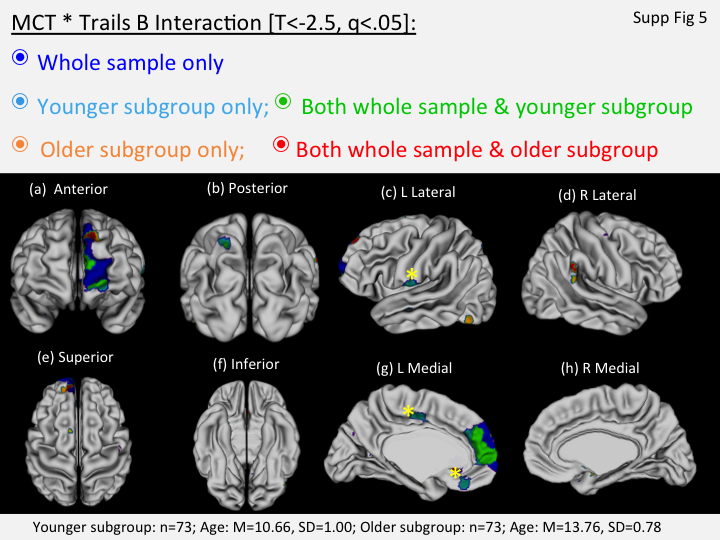

Supplement: Supplementary Figure S1 — Regions in which there were main effects of mean cortical thickness, Trails B performance, and their interaction in predicting vertex thickness. This figure supplements Figure 1 in the main document. Linear regression analyses predicting cortical thickness at each vertex in both hemispheres were run in the complete sample (n = 146) of participants in order to evaluate the effects of mean cortical thickness, Trails B age-adjusted scores, and their interaction. The regression equation was as follows: Cortical thickness (vertex j) = Intercept + ß1(MCT) + ß2(Trails B time) + ß3(MCT*Trails B). Note that for these analyses, the vertex-level dependent variables and MCT were age and sex standardized. (See Materials and Methods for details). The Trails B variables were age-standardized. T-statistics associated with each of the effects in the regression equation were corrected for multiple comparisons using a False Discovery Rate adjustment. Only those vertices with qs < 0.05 (associated with a T-threshold of 2.5) are displayed in this figure in (A–H). Vertices in purple are those in which a main effect of MCT was found; vertices in blue are those in which main effects of MCT and Trails B were found such that thinner cortex was associated with better performance; vertices in turquoise green are those in which main effects of MCT and Trails B were found such that thicker cortex was associated with better performance; lastly, vertices in yellow are those in which an MCT*Trails B interaction was found such that greater coupling was associated with better performance. [file Presentation1.ZIP › 86138_Lee_Figure_8.TIFF]

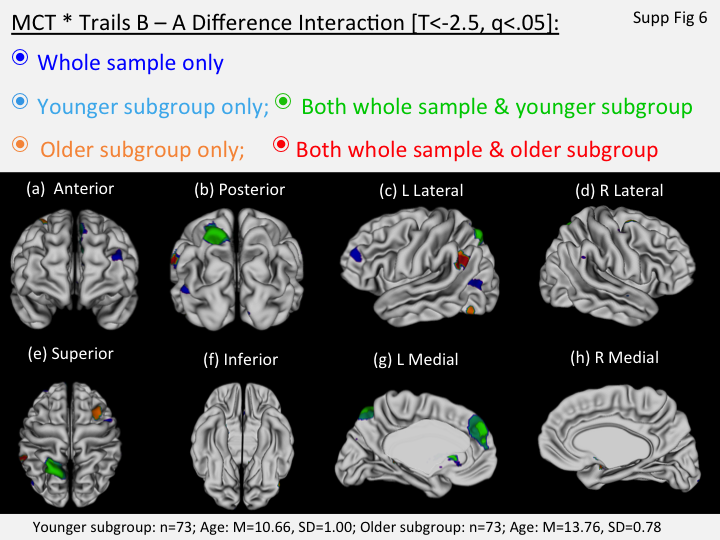

Supplement: Supplementary Figure S1 — Regions in which there were main effects of mean cortical thickness, Trails B performance, and their interaction in predicting vertex thickness. This figure supplements Figure 1 in the main document. Linear regression analyses predicting cortical thickness at each vertex in both hemispheres were run in the complete sample (n = 146) of participants in order to evaluate the effects of mean cortical thickness, Trails B age-adjusted scores, and their interaction. The regression equation was as follows: Cortical thickness (vertex j) = Intercept + ß1(MCT) + ß2(Trails B time) + ß3(MCT*Trails B). Note that for these analyses, the vertex-level dependent variables and MCT were age and sex standardized. (See Materials and Methods for details). The Trails B variables were age-standardized. T-statistics associated with each of the effects in the regression equation were corrected for multiple comparisons using a False Discovery Rate adjustment. Only those vertices with qs < 0.05 (associated with a T-threshold of 2.5) are displayed in this figure in (A–H). Vertices in purple are those in which a main effect of MCT was found; vertices in blue are those in which main effects of MCT and Trails B were found such that thinner cortex was associated with better performance; vertices in turquoise green are those in which main effects of MCT and Trails B were found such that thicker cortex was associated with better performance; lastly, vertices in yellow are those in which an MCT*Trails B interaction was found such that greater coupling was associated with better performance. [file Presentation1.ZIP › 86138_Lee_Figure_9.TIFF]

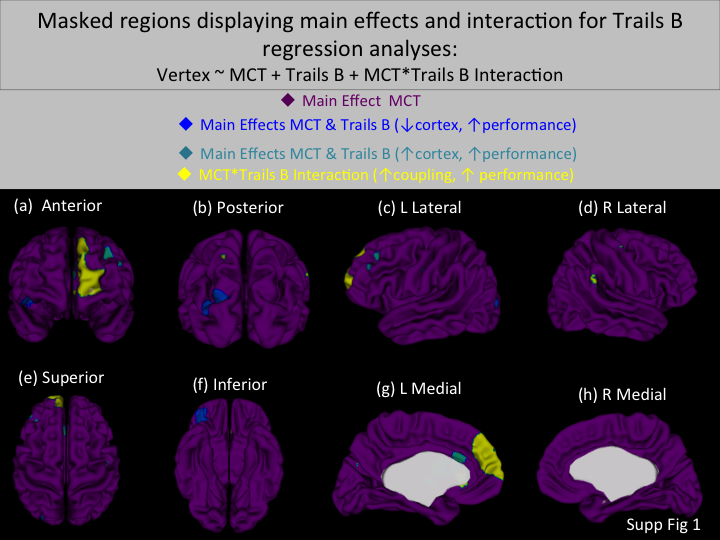

Supplement: Supplementary Figure S1 — Regions in which there were main effects of mean cortical thickness, Trails B performance, and their interaction in predicting vertex thickness. This figure supplements Figure 1 in the main document. Linear regression analyses predicting cortical thickness at each vertex in both hemispheres were run in the complete sample (n = 146) of participants in order to evaluate the effects of mean cortical thickness, Trails B age-adjusted scores, and their interaction. The regression equation was as follows: Cortical thickness (vertex j) = Intercept + ß1(MCT) + ß2(Trails B time) + ß3(MCT*Trails B). Note that for these analyses, the vertex-level dependent variables and MCT were age and sex standardized. (See Materials and Methods for details). The Trails B variables were age-standardized. T-statistics associated with each of the effects in the regression equation were corrected for multiple comparisons using a False Discovery Rate adjustment. Only those vertices with qs < 0.05 (associated with a T-threshold of 2.5) are displayed in this figure in (A–H). Vertices in purple are those in which a main effect of MCT was found; vertices in blue are those in which main effects of MCT and Trails B were found such that thinner cortex was associated with better performance; vertices in turquoise green are those in which main effects of MCT and Trails B were found such that thicker cortex was associated with better performance; lastly, vertices in yellow are those in which an MCT*Trails B interaction was found such that greater coupling was associated with better performance. [file Presentation1.ZIP › 86138_Lee_Figure_4.TIFF]

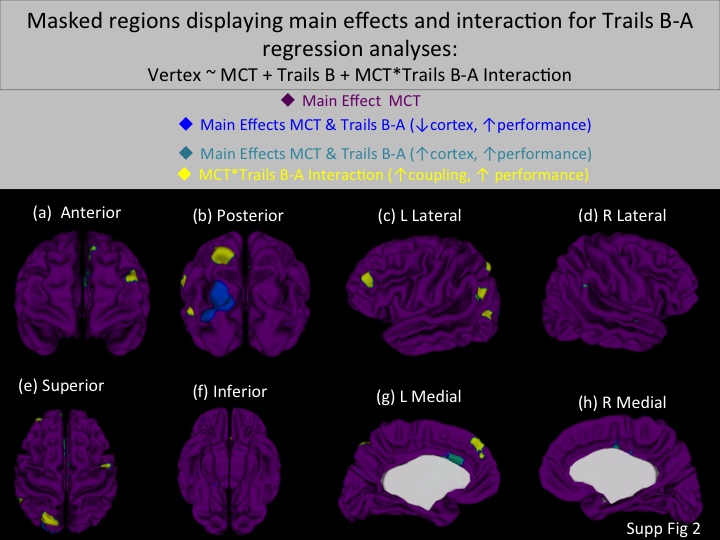

Supplement: Supplementary Figure S1 — Regions in which there were main effects of mean cortical thickness, Trails B performance, and their interaction in predicting vertex thickness. This figure supplements Figure 1 in the main document. Linear regression analyses predicting cortical thickness at each vertex in both hemispheres were run in the complete sample (n = 146) of participants in order to evaluate the effects of mean cortical thickness, Trails B age-adjusted scores, and their interaction. The regression equation was as follows: Cortical thickness (vertex j) = Intercept + ß1(MCT) + ß2(Trails B time) + ß3(MCT*Trails B). Note that for these analyses, the vertex-level dependent variables and MCT were age and sex standardized. (See Materials and Methods for details). The Trails B variables were age-standardized. T-statistics associated with each of the effects in the regression equation were corrected for multiple comparisons using a False Discovery Rate adjustment. Only those vertices with qs < 0.05 (associated with a T-threshold of 2.5) are displayed in this figure in (A–H). Vertices in purple are those in which a main effect of MCT was found; vertices in blue are those in which main effects of MCT and Trails B were found such that thinner cortex was associated with better performance; vertices in turquoise green are those in which main effects of MCT and Trails B were found such that thicker cortex was associated with better performance; lastly, vertices in yellow are those in which an MCT*Trails B interaction was found such that greater coupling was associated with better performance. [file Presentation1.ZIP › 86138_Lee_Figure_5.TIFF]

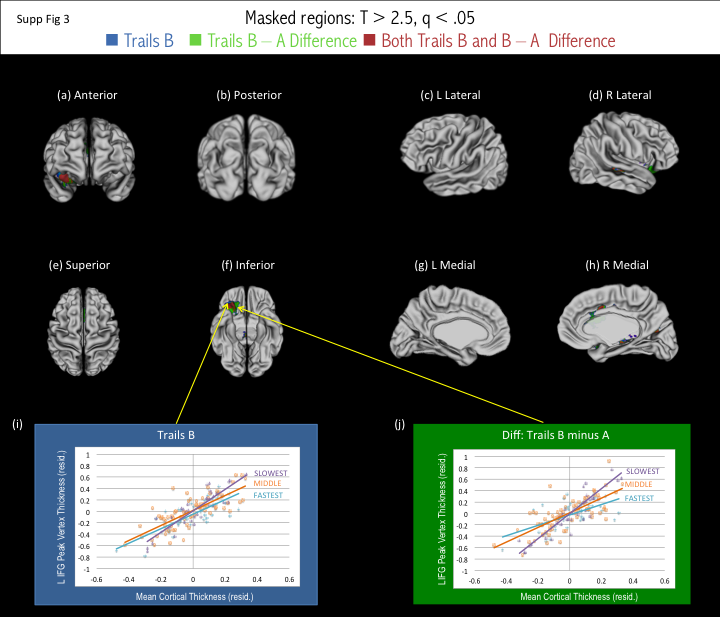

Supplement: Supplementary Figure S1 — Regions in which there were main effects of mean cortical thickness, Trails B performance, and their interaction in predicting vertex thickness. This figure supplements Figure 1 in the main document. Linear regression analyses predicting cortical thickness at each vertex in both hemispheres were run in the complete sample (n = 146) of participants in order to evaluate the effects of mean cortical thickness, Trails B age-adjusted scores, and their interaction. The regression equation was as follows: Cortical thickness (vertex j) = Intercept + ß1(MCT) + ß2(Trails B time) + ß3(MCT*Trails B). Note that for these analyses, the vertex-level dependent variables and MCT were age and sex standardized. (See Materials and Methods for details). The Trails B variables were age-standardized. T-statistics associated with each of the effects in the regression equation were corrected for multiple comparisons using a False Discovery Rate adjustment. Only those vertices with qs < 0.05 (associated with a T-threshold of 2.5) are displayed in this figure in (A–H). Vertices in purple are those in which a main effect of MCT was found; vertices in blue are those in which main effects of MCT and Trails B were found such that thinner cortex was associated with better performance; vertices in turquoise green are those in which main effects of MCT and Trails B were found such that thicker cortex was associated with better performance; lastly, vertices in yellow are those in which an MCT*Trails B interaction was found such that greater coupling was associated with better performance. [file Presentation1.ZIP › 86138_Lee_Figure_6.TIFF]

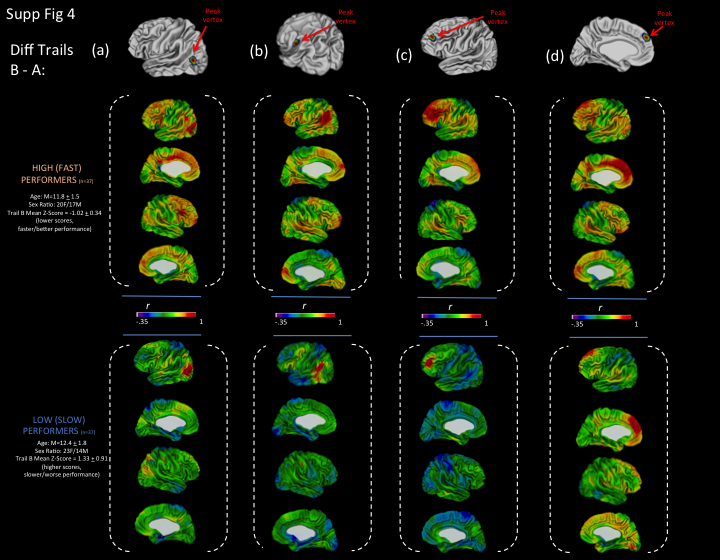

Supplement: Supplementary Figure S1 — Regions in which there were main effects of mean cortical thickness, Trails B performance, and their interaction in predicting vertex thickness. This figure supplements Figure 1 in the main document. Linear regression analyses predicting cortical thickness at each vertex in both hemispheres were run in the complete sample (n = 146) of participants in order to evaluate the effects of mean cortical thickness, Trails B age-adjusted scores, and their interaction. The regression equation was as follows: Cortical thickness (vertex j) = Intercept + ß1(MCT) + ß2(Trails B time) + ß3(MCT*Trails B). Note that for these analyses, the vertex-level dependent variables and MCT were age and sex standardized. (See Materials and Methods for details). The Trails B variables were age-standardized. T-statistics associated with each of the effects in the regression equation were corrected for multiple comparisons using a False Discovery Rate adjustment. Only those vertices with qs < 0.05 (associated with a T-threshold of 2.5) are displayed in this figure in (A–H). Vertices in purple are those in which a main effect of MCT was found; vertices in blue are those in which main effects of MCT and Trails B were found such that thinner cortex was associated with better performance; vertices in turquoise green are those in which main effects of MCT and Trails B were found such that thicker cortex was associated with better performance; lastly, vertices in yellow are those in which an MCT*Trails B interaction was found such that greater coupling was associated with better performance. [file Presentation1.ZIP › 86138_Lee_Figure_7.TIFF]
